# Meteorin is upregulated in reactive astrocytes and functions as a negative feedback effector in reactive gliosis

**DOI:** 10.3892/mmr.2015.3610

**Published:** 2015-04-14

**Authors:** HYE SHIN LEE, SOON-HEE LEE, JONG-HO CHA, JI HAE SEO, BUM JU AHN, KYU-WON KIM

**Affiliations:** 1 SNU-Harvard Neurovascular Protection Research Center, Department of Pharmacy, College of Pharmacy and Research Institute of Pharmaceutical Sciences, Seoul National University, Seoul 151-742, Republic of Korea; 2 Department of Molecular Medicine and Biopharmaceutical Sciences, Graduate School of Convergence Science and Technology and College of Medicine or College of Pharmacy, Seoul National University, Seoul 151-742, Republic of Korea

**Keywords:** meteorin, reactive gliosis, astrocyte, photothrombotic ischemia

## Abstract

Reactive gliosis is a glial response to a wide range of central nervous system insults, which results in cellular and molecular changes to resting glial cells. Despite its fundamental effect on neuropathologies, the identification and characterization of the molecular mechanisms underlying this process remain to be fully elucidated. The aim of the present study was to analyze the expression profile and functions of the astrocytic neurotrophic factor, meteorin, in the progression of reactive gliosis. A mouse model of photothrombotic ischemia, and a primary astrocyte culture were used in the present study. Reverse transcription quantitative polymerase chain reaction, western blotting and immunofluorescence staining were performed to examine the expression levels of meteorin and reactive gliosis markers. Increased expression levels of meteorin were observed in reactive astrocytes in a photothrombotic ischemia mouse model, as well as in cultured astrocytes, which were stimulated by transforming growth factor-β1. Exogenous treatment of the astrocytes with meteorin did not induce janus kinase-signal transducer and activator of transcription 3 signaling, however, silencing the expression of meteorin in the astrocytes resulted in an upregulation of reactive astrocyte markers, including glial fibrillary acidic protein and S100β, indicating that endogenous meteorin is required for the maintenance of astrocytic homeostasis. These results suggested a novel role for meteorin as a negative feedback effector in reactive gliosis.

## Introduction

Astrocytes are a type of macroglial cell, and are the most abundant type of cell in the central nervous system (CNS) (1). Astrocytes were previously considered to have predominantly supportive roles to assist in appropriate neuronal functions; however, with the development of molecular neuroscience, studies have revealed several active roles of astrocytes, including the regulation of blood flow (2,3), involvement in synaptic functions (1,4) and maintenance of the blood-brain barrier (BBB) integrity (5).

Reactive gliosis is an astrocytic response to a wide range of CNS pathologies, which results in morphological and molecular changes in the resting glia. Although the extent of the changes in astrocytes due to astrogliosis vary with the nature and severity of an insult, there are certain changes that astrocytes commonly undergo in response to all forms of CNS insult, including hypertrophy, proliferation and the upregulation of molecules, including glial fibrillary acidic protein (GFAP) and S100β (6). In certain severe case of CNS injury, a physical barrier, referred to as a ‘glial scar’ is formed by glial cells undergoing reactive gliosis, to isolate the damaged tissue from the healthy region and to prevent a cascade of inflammation, caused by factors released from the lesion site (7). However, it is difficult to categorize the effect of the glial scar as beneficial or harmful in terms of clinical outcome, since this tight physical barrier also inhibits the regeneration of damaged neurons at a later stage of injury (8–10). Therefore, determining the mechanisms by which reactive gliosis is regulated may improve clinical outcomes, however, the molecular and cellular mechanisms underlying the initiation, progression and resolution of this process remain to be fully elucidated.

Meteorin is a secreted protein, which was initially screened as a retinoic-acid-responding molecule (11). In the developing rodent CNS, meteorin is highly expressed in the neuroepithelium and remains in subpopulations of the glia, including the cortical astrocytes, following completion of CNS development (11,12). Our previous study reported that meteorin promotes glial differentiation in neural stem cells (NSCs) via activation of the janus kinase (Jak)-signal transducer and activator of trascription 3 (STAT3) signaling pathway (12), and that astrocytic meteorin promotes the maturation of the BBB by stimulating endothelial cells (13). Regarding its involvement in CNS pathologies, previous studies have reported on the neuroprotective effects of meteorin in rat models of neuropathic pain and in quinolic acid-induced neuropathology (14,15). In addition, a previous study identified a novel function of meteorin in promoting neuroblast migration in a stroke model (16). Despite emerging evidence regarding the crucial functions of meteorin in neuropathologies, its role in the glial response to CNS insults remains to be elucidated.

The predominant source of meteorin in the adult brain are astrocytes, and meteorin is known to promote glial cell differentiation and the expression of GFAP in NSCs (12,17); therefore, the present study aimed to demonstrate that meteorin was involved in the reactive gliosis process. The expression levels of meteorin were analyzed in reactive astrocytes from a photothrombotic (PT) ischemia mouse model, as well as following stimulation of transforming growth factor (TGF)-β, which led to the activation of astrocytes *in vitro*. A small interfering RNA (siRNA)-mediated meteorin-knockdown was performed to identify the role of meteorin in reactive gliosis.

## Materials and methods

### Induction of PT ischemia in vivo

C57BL/6 mice (3–6 months old) were purchased from Samtako (Daejeon, Korea) and PT ischemia was induced, as described previously (18). Briefly, under deep anesthesia by intraperitoneal injection of Zoletil^®^ (30 mg/kg) and Rompun^®^ (10 mg/kg) (Virbac, Carros, France), rose bengal photosensitive dye (Sigma-Aldrich; 0.1 ml of a 10% solution/25 g body weight) was injected into the tail vein and allowed to circulate for 5 min. The skull was then exposed to a cold light source (150 W, 1 mm diameter; Zeiss FL1500 LCD; Carl Zeiss AG) for 20 min, 2.5 mm to the left and 2.5 mm to the back of the bregma. A total of 7 days following PT induction, the mice were deeply anesthesized and cardiac tissue was perfused with PBS, followed by 4% paraformaldehyde/PBS solution for immunohistochemical analysis. Alternatively, fresh brain tissue was isolated, without cardiac perfusion, and the lesion sites were dissected to isolate total RNA. Contralateral hemisphere tissue was considered as a control.

The animal experiments of the present study were approved by the Committee for Care and Use of Laboratory Animals at the Seoul National University (Seoul, Korea), according to the Guide for Animal Experiments edited by the Korean Academy for Medical Sciences. The mice were maintained in a specific pathogen-free room in the animal-housing facilities at Seoul National University under a 12-h dark/light cycle with a provision of chow and water *ad libitum*.

### Cell culture and purification of recombinant meteorin

Primary cultures of mouse cortical astrocytes were prepared from the brains of 2 day-old mice (Samtako). The mice were sacrificed by decapitation, and the brains were isolated. The cerebral cortices were dissected from the mouse brains and the meninges were removed. The cortices were then diced into 1–2 mm sections and incubated in 0.25% trypsin (Life Technologies, Carlsbad, CA, USA) and 20 mg/ml DNase I (Sigma-Aldrich, St. Louis, MO, USA) at 37°C for 20 min. The sections were then dissociated into single cells by pipetting. The cells were plated and cultured in Dulbecco’s modified Eagle’s medium (DMEM; Life Technologies), supplemented with 10% fetal bovine serum (FBS; Life Technologies) and 1% penicillin/streptomycin (Life Technologies). After 7–10 days of culture, the cells were agitated at 250 rpm for 16 h, in order to remove the microglia and oligodendrocytes. Once the cells had reached 80% confluence they were treated with the indicated amount of TGF-β1 (10 or 50 ng/ml; PeproTech, Rocky Hill, NJ, USA) for 24 h.

Primary NSCs were cultured from E13.5 mouse embryos, as described previously (12). Briefly, embryos were dissected from pregnant female mice (Samtako) and neurospheres were allowed to form in complete neurobasal medium (Invitrogen Life Technologies) containing 2% B27 (Invitrogen Life Technologies), human epidermal growth factor (20 ng/ml, R&D Systems, Minneapolis, MN, USA), and basic fibroblast growth factor (10 ng/ml, Invitrogen Life Technologies) for 4 days, by plating neuroepithelial cells on non-coated culture dishes. For *in vitro* differentiation, the neurospheres were dissociated with trypsin-EDTA and plated onto poly-L-ornithine (Sigma-Aldrich)-coated dishes in complete medium. The neurospheres were treated with 200 ng/ml recombinant meteorin for 48 h following an overnight deprivation of growth factors.

Recombinant mouse meteorin was purified from the conditioned medium of CHO-K1 Chinese hamster ovary cells (Korean Cell Line Bank, Seoul, Korea) stably expressing meteorin tagged with myc-His6 at C-terminus, as described previously (13). CHO-K1 cells were maintained in DMEM supplemented with 10% FBS at 37°C in a humidified atmosphere containing 5% CO_2_.

### siRNA preparation and transfection

The following siRNAs were synthesized and were used to target mouse meteorin: siMeteorin #1, 5′-GTTCAGCCGTGTCTATTCA-3′; and siMeteorin #2, 5′-GTCTTCGCTGAACGTATGA-3′. Non-targeting siRNAs were used as a control (GE Dharmacon, Lafayette, CO, USA). The astrocytes were transfected with the siRNAs using oligofectamine (Invitrogen Life Technologies, Carlsbad, CA, USA) once they had reached ~50% confluence.

### Western blot analysis and reverse transcription-quantitative polymerase chain reaction (RT-qPCR)

The astrocytes were lysed using 1X cell lysis buffer (Cell Signaling Technology Inc., Beverly, MA, USA). Protein concentration was determined using bicinchoninic acid protein assay kit (Invitrogen Life Technologies). The protein samples (40 *μ*g) were then separated by 12% SDS-PAGE and transferred to nitrocellulose membranes (GE Healthcare Life Sciences, Piscataway, NJ, USA). After blocking with 5% skim milk/phosphate-buffered saline (PBS) with 0.1% Tween-20, the membranes were immunoblotted with the following antibodies overnight at 4°C: Rabbit anti-phospho-STAT3 (Tyr705) (1:2,000 cat. no. 9131; Cell Signaling Technology, Inc.), rabbit anti-STAT3 (1:5,000; cat. no. sc-482; Santa Cruz Biotechnology, Inc., Dallas, TX, USA), rabbit anti-GFAP (1:1,000; cat. no. s0334; DAKO, Glostrup, Denmark) and rabbit anti-actin (1:10,000; cat. no. A2668; Sigma-Aldrich). The membranes were then probed with anti-rabbit secondary antibodies conjugated to horseradish peroxidase (1:5,000; cat. no. HAF008; R&D Systems) for 1 h at room temperature, and proteins were visualized using an enhanced chemiluminescence system (Intron Biotechnology, Gyeonggi-do, Korea). Band intensities were quantified using MultiGauge v.3.0 software (Fujifilm, Tokyo, Japan).

RT-qPCR was performed, as described previously (12). Briefly, total RNA was isolated using TRIzol^®^ reagent (Invitrogen Life Technologies), according to the manufacturer’s instructions. To isolate total RNA from brain tissue, the samples were homogenized in TRIzol by passing through a 30 gauge needle of a 1 ml syringe. First-stranded cDNA was synthesized from 2 *μ*g total RNA using a murine leukemia virus reverse transcriptase (Promega Corporation, Madison, WI, USA). A total of 2 *μ*l cDNA was then amplified by PCR using the following thermocycling conditions: 95°C for 30 sec, 55°C for 30 sec, 72°C for 60 sec, 25 cycles on a thermal cycler (Applied Biosystems, Foster City, CA, USA). The PCR products were visualized following separation by 1.5% agarose gel electrophoresis and the band intensities were quantified using MultiGauge v.3.0 software (Fujifilm). The following primers (Bioneer Corporation, Daejeon, Korea) were used to amplify each gene: Meteorin, forward 5′-ATGCTGGTAGCCACGCTTCTTT-3′ and reverse 5′-GTCCAGTGCCATCTCACATGGG-3′), *Gfap*, forward 5′-GGCCGGGGCGCTCAA-3′ and reverse 5′-GCCGACTCCCGCGCAT-3′), *S100β*, forward 5′-GGTTGCCCTCATTGATGTCT-3′, and reverse 5′-GTCCAGCGTCTCCATCACTT-3′ and *Gapdh*, forward 5′-ACCACAGTCCATGCCATCAC-3′ and reverse 5′-TCCACCACCCTGTTGCTGTA-3′).

### Immunohistochemistry

Coronal brain sections (30 *μ*m thickness) isolated from mice 7 days following PT induction were prepared using a cryotome and collected in PBS. Free-floating sections were then permeabilized with PBS-0.25% Triton-X, blocked with 5% FBS, and treated with the following primary antibodies: Rabbit anti-GFAP (1:750; DAKO), goat anti-meteorin (1:100; R&D Systems, Minneapolis, MN, USA), and mouse anti-S100β (1:500; Sigma-Aldrich) in 5% FBS overnight at 4°C. Following extensive washing with PBS-0.25% Triton-X, the sections were incubated with Alexa Fluor-conjugated secondary antibodies (1:100) for 2 h at room temperature, and the tissues were mounted using FluorSave™ Reagent (EMD Millipore, Billerica, MA, USA). Images of the tissues were captured using an M200 ApoTome microscope (Carl Zeiss AG, Oberkochen, Germany) and a LSM700 confocal microscope (Carl Zeiss AG).

### Statistical analysis

The data are expressed as the mean ± standard error of the mean. Statistical significance was determined using an unpaired two-tailed Student’s t-test in Microsoft Excel 2010 (Microsoft Corporation, Redmond, WA, USA). P<0.05 was considered to indicate a statistically significant difference.

## Results

### Meteorin is upregulated in reactive astrocytes following PT insult

**Figure 1:**
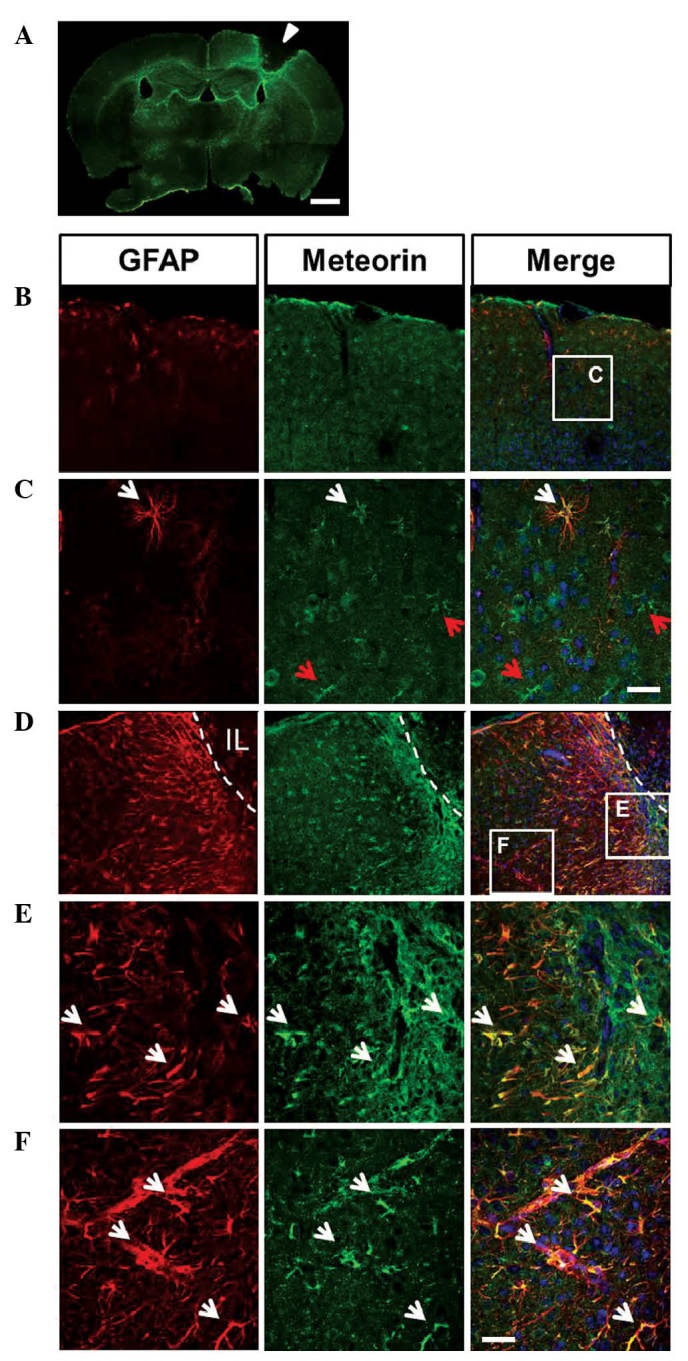
Upregulated expression of meteorin in reactive astrocytes in the peri-infarct cortex. (A) GFAP staining of mouse brain sections 7 days after PT induction revealed marked reactive gliosis surrounding the infarct lesion (arrow head). (B) Double immunofluorescence staining of GFAP and meteorin in the contralateral hemisphere. (C) Higher magnification images revealed that meteorin was expressed in the GFAP-positive astrocytes (white arrow) and the GFAP-negative astrocytes (red arrows). (D) Double immunofluorescence staining of GFAP (red) and meteorin (green) in the peri-infarct region 7 days after PT induction. Reactive astrocytes presented exhibited increased expresseion levels of GFAP and meteorin. Dashed lines indicate the boundary of the infarct lesion (IL). (E) Higher magnification images of the glial scar region revealed hypertrophic reactive astrocytes markedly expressing meteorin. Arrows indicate representative cells expressing GFAP and meteorin. (F) Higher magnification images of a relatively distal region from the infarct reveal a lesser extent of meteorin upregulation in the reactive astrocytes in this region. Arrows indicate representative cells double labeled for GFAP and meteorin. Scale bars=2 mm in (A) and 50 *μ*m in (B–F). GFAP, glial fibrillary acidic protein; PT, photothrombotic ischemia.

To determine the functions of meteorin in reactive gliosis, the present study examined its expression in reactive astrocytes using immunofluorescent staining. The PT ischemia mouse model was used as a brain injury model, in which photochemical occlusion of the irradiated vessels with secondary tissue ischemia is induced by photosensitive dyes like rose-bengal and a local irradiation of cold light through the skull (19). At 7 days following PT induction, infarct lesions were observed and a glial scar surrounding the lesions had formed, as revealed by GFAP staining (Fig. 1A). In the cortex of the contralateral hemisphere, in which the majority of astrocytes are at a resting state, only a subset of astrocytes, predominantly beneath the meningeal epithelium or surrounding blood vessels exhibited GFAP immunoreactivity. Double immunostaining of GFAP and meteorin demonstrated that, not only GFAP-positive, but also GFAP-negative astrocytes exhibited weak expression levels of meteorin (Fig. 1B and C). This pattern of meteorin staining in the normal brain was concordant with the findings of our previous study and of others (11,13,17).

**Figure 2:**
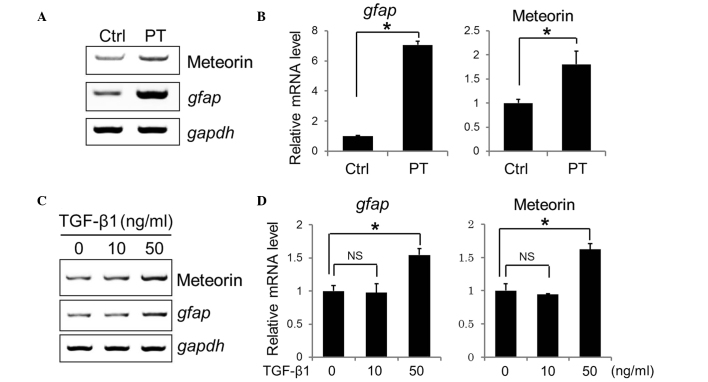
mRNA expression levels of *meteorin* are increased in reactive astrocytes from PT tissue and following *in vitro* TGF-β1 stimulation of astrocyte activation. (A and B) RT-qPCR revealed increased expression levels of *meteorin* and *Gfap* in PT tissues. (B) Graphs of the quantification of the expression levels of *Gfap* (left) and *meteorin* (right) normalized to gapdh levels. n=3, Data are expressed as the mean ± standard error of the mean. ^*^P<0.05, compared with the control. (C and D) RT-qPCR revealed increased expression levels of *meteorin* and *Gfap* following TGF-β1 stimulation for 24 h in the cultured astrocytes. (D) Graphs of the quantification of the expression levels of *Gfap* (left) and *meteorin* (right) normalized to gapdh levels. n=3. Data are expressed as the mean ± standard error of the mean. ^*^P<0.05, compared with the untreated cells. NS, not significant; Ctrl, control; PT photothrombotic ischemia; GFAP, glial fibrillary acidic protein; TGF-β1, transforming growth factor-β1; RT-qPCR, reverse transcription-quantitative polymerase chain reaction.

By contrast, a marked increase in the number of GFAP-positive cells was observed in the cortex exhibiting infarct lesions, and these cells exhibited significantly more marked GFAP staining (Fig. 1A and D–F). Furthermore, a typical glial scar formed at the edge of the infarct lesion, where the GFAP-positive cells had built a dense network. The meteorin staining pattern in the infarct side was similar to that of GFAP, being more marked in the glial scar region and exhibiting a gradual decrease in the areas distant from the infarct lesion (Fig. 1D–F). To further confirm the upregulation of meteorin induced by PT, the cortex of the infracted hemisphere and the contralateral hemisphere were dissected, and total RNAs were extracted to compare the mRNA expression levels of *meteorin* and *Gfap*. RT-qPCR revealed a 1.7-fold increase in the expression of *meteorin* and a 7-fold increase in *Gfap* (Fig. 2A and B). These data indicated that the expression of *meteorin* was increased in the reactive astrocytes, activated by PT insult.

### Expression of meteorin is increased in response to TGF-β stimulation in vitro

The activation of astrocytes can be triggered by various factors, including cytokines and nitric oxide, which are produced by microglia and other immune cells in infarct lesions (7). To determine whether the *in vitro* stimulation of astrocyte activation leads to changes in the expression of *meteorin*, primary mouse cortex astrocytes were cultured and treated with increasing doses of TGF-β1, which is a well-known reactive gliosis-promoting factor (20). Treatment with recombinant TGF-β1 at a concentration of 50 ng/ml resulted in ~1.5-fold and 1.7-fold increases in the mRNA expression levels of *Gfap* and *meteorin*, respectively. However, no effects were observed following treatment of the cells with 10 ng/ml TGF-β1 (Fig. 2C and D).

### Meteorin activates different signaling pathways in astrocytes and NSCs

**Figure 3:**
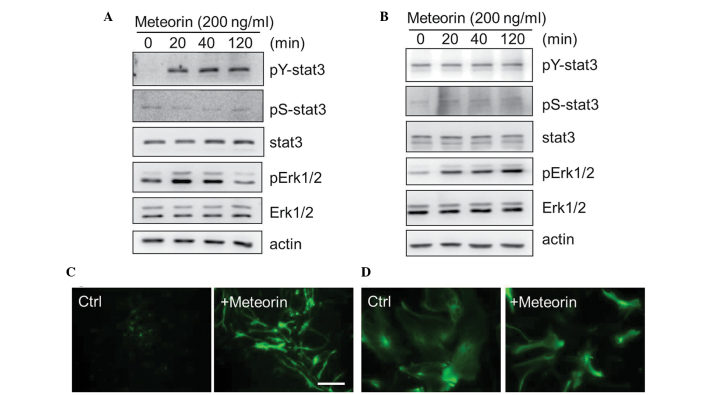
Exogenous meteorin treatment does not induce Jak-STAT3 signaling in cultured astrocytes. (A and B) Western blot anaylses of the activation of signaling proteins following treatment of either (A) NSCs or (B) primary mouse astrocytes with meteorin (200 ng/ml) at the indicated time-points. Tyrosine phosphorylation of STAT3 was induced by meteorin in the NSCs, but not in the astrocytes, whereas Erk1/2 phosphorylation was observed in the two types of cell. (C and D) Immunocytochemical GFAP staining of (C) cultured NSCs and (D) astrocytes (D) in the absence (ctrl) or presence of meteorin. Meteorin promoted the formation of GFAP-positive astrocytes in the (C) NSCs but had no effect on the (D) astrocytes. Scale bar=50 *μ*m Ctrl, control; Jak janus kinase; STAT3, signal transducer and activator of transcription 3; NSC, neural stem cells; GFAP, glial fibrillary acidic protein; Erk, extracellular signal-regulated kinase.

Our previous study demonstrated that the Jak-STAT3 pathway is a downstream signaling unit of meteorin in neural progenitor cell differentiation (12). Therefore, the present study aimed to analyze the effects of recombinant meteorin treatment on astrocyte activation in comparison to its effects on NSCs. Concordant with the findings of our previous report, treatment of the NSCs, from embryonic mouse brain tissues cultured with recombinant meteorin (200 ng/ml) induced the tyrosine phosphorylation of STAT3 and activation of extracellular signal-regulated kinase (Erk)1/2, as early as 20 min after treatment (Fig. 3A). The phosphorylation of STAT3 by meteorin was not detected in the astrocytes, although subsequent Erk1/2 activation was observed (Fig. 3B). The NSCs and astrocytes were also treated with recombinant meteorin for 3 days, followed by GFAP staining, to determine whether meteorin treatment increased the expression of GFAP. Treatment of NSCs with meteorin resulted in an increase in the number of GFAP-positive cells, possibly due to hyperactivation of STAT3 signaling (Fig. 3C). However, no significant changes in GFAP staining were observed in the astrocyte cultures treated with the same dose of meteorin (Fig. 3D). These results indicated that meteorin activated different signaling pathways depending on the cell type, but appears unlikely to act as an inducer of reactive gliosis.

### Silencing the expression of meteorin results in upregulation of reactive astrocyte markers

**Figure 4:**
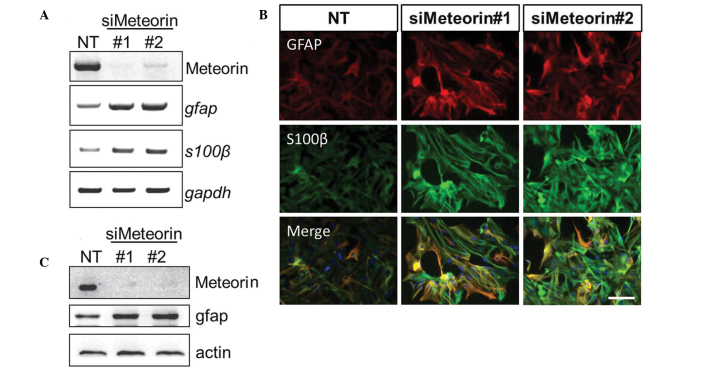
Silencing the expression of meteorin leads to upregulation of reactive astrocyte molecular markers. (A) RT-qPCR of the relative expression levels of transcripts for the indicated genes. Astrocytes were transfected with two siRNAs targeting meteorin (#1 and #2), or non-targeting siRNAs (NT) as a control, and RT-qPCR was performed 24 h post-transfection. (B) Immunocytochemistry revealed increased GFAP (red) and S100β (green) staining in the meteorin-knockdown astrocytes. Scale bar=50 *μ*m. (C) Western blotting demonstrated increased protein expression levels of GFAP in the meteorin-knockdown astrocytes. The cells were lysed 72 h after siRNA transfection and actin was used as a loading control. siRNA, small interfering RNA; RT-qPCR, reverse transcription-quantitative polymerase chain reaction; GFAP, glial fibrillary acidic protein.

To further analyze the role of meteorin in astrocyte activation, the gene expression of meteorin was silenced using siRNAs specifically targeting mouse meteorin. Efficient knockdown of the expression of metorin in astrocytes using two different siRNAs was confirmed by RT-qPCR and western blotting. A correlation between the expression of meteorin and astrocyte activation was determined by comparing the expression levels of GFAP and S100β. The two meteorin-targeting siRNAs resulted in increased mRNA and protein expression levels of GFAP and S100β, determined by RT-qPCR and western blotting, respectively (Fig. 4A and C). In addition, immunocytochemical staining for GFAP and S100β was more marked in the astrocytes transfected with meteorin-targeting siRNAs (Fig. 4B). These results suggested the possible function of meteorin in a negative feedback loop in reactive gliosis, during which glial activation is resolved and cells revert to their resting state.

## Discussion

The present study analyzed the expression profile and functions of meteorin in reactive gliosis in the mouse brain. The results demonstrated: i) Meteorin was expressed in cortical astrocytes and its expression was upregulated in reactive astrocytes; ii) exogenous application of meteorin to astrocytes did not activate STAT3 signaling; and iii) silencing the expression of meteorin in astrocytes led to the upregulation of reactive astrocyte markers.

In the majority of CNS injuries, astrocytes undergo reactive gliosis, which can be beneficial and detrimental. Although a physical barrier, by formation of a glial scar, is the primary defense against damage, the beneficial effects of reactive gliosis are also mediated by the secretion of soluble factors (20). For example, the expression of ciliary neurotrophic factor is known to be increased in reactive astrocytes following ischemic insult, and its neuroprotective effects have been demonstrated in a wide range of animal models of CNS injury (21–23).

It is noteworthy that meteorin has been reported to have neuroprotective effects in various neuropathologiacal models (14,15). A possible scenario, based on previous studies (14,15,20) and the present study, is that cytokines, including TGF-β1, trigger reactive gliosis to promote the production of meteorin by astrocytes, and meteorin subsequently acts on neurons to regenerate axons in a paracrine manner. In addition, meteorin may signal to astrocytes in an autocrine manner as a negative feedback factor, which in turn leads to the resolution of reactive gliosis. Notably, one of the beneficial effects of reactive gliosis is to restore the BBB following brain injury (24,25), and our previous study identified meteorin as a maturation factor for brain vascular development (13). Therefore, analyzing the effects of meteorin administration *in vivo* following brain injury, in terms of neuroprotection, reactive gliosis and BBB integrity is worthwile.

Since the first report regarding meteorin in 2004 (11), numerous lines of investigation by independent groups have uncovered its novel functions and characteristics (11–16). However, the cellular receptor(s) of meteorin remain to be elucidated. In our previous study, the Jak-STAT3 pathway was found to be involved in the downstream signaling of meteorin in NSC differentiation. In the present study, meteorin did not activate the same pathway in astrocytes, indicating another layer of complexity in meteorin signaling. One possible explanation is that there is more than one meteorin receptor, which is differentially expressed in distinct cell types and they activate distinct signaling pathways; however, additional investigations are required to confirm this hypothesis.
